# Prevalence of trachoma and associated factors in students from the Jequitinhonha Valley, Minas Gerais, Brazil

**DOI:** 10.1590/0037-8682-0056-2020

**Published:** 2020-10-21

**Authors:** Evanildo José da Silva, Daniela Porto Pereira, João Octávio Augusto Murta Ambrózio, Laiara Morais Barboza, Vivian Ladeira Fonseca, Antônio Prates Caldeira

**Affiliations:** 1 Universidade Federal dos Vales do Jequitinhonha e Mucuri, Departamento de Medicina, Diamantina, MG, Brasil.; 2 Universidade Estadual de Montes Claros, Departamento de Medicina, Montes Claros, MG, Brasil.

**Keywords:** Chlamydia trachomatis, Neglected disease, Epidemiology

## Abstract

**INTRODUCTION:**

Trachoma is the leading cause of blindness in the world, especially in undeveloped countries, due to its association with poor socioeconomic and sanitation conditions. This study aimed to estimate the prevalence of trachoma among students from the Jequitinhonha Valley, Minas Gerais, one of the poorest regions in Brazil, and to identify associated factors.

**METHODS:**

This is a cross-sectional study that utilized clinical evaluation and a socioeconomic questionnaire applied to a random and representative sample of elementary school students from the Jequitinhonha Valley, Minas Gerais, Brazil. Participants underwent conjunctival scraping and direct immunofluorescence was used to confirm the presence of the bacteria. Five or more elementary bodies in the conjunctival scrape was considered a positive result. In the study, 36.6% positive samples were detected. A culture of the conjunctival scrape, considered to be the "gold standard", was not performed due to cost and complexity. Bivariate analyses were performed, followed by binary logistic regression analysis to define the associated variables.

**RESULTS:**

In the present study, 478 students comprised the sample. The prevalence of trachoma was 6.3% and was higher among students who lived in unfinished houses (no plastering, painting, flooring, and unfinished bathrooms) (OR, 2.27; 95% CI, 1.12-6.48) without sewage systems (OR = 9.49; 95% CI = 3.52-25.60) and studied in rural areas (OR, 3.37; 95% CI, 1.53-7.35).

**CONCLUSIONS:**

The prevalence of trachoma among the students aged 7 to 16 years old, from public and private schools, is not negligible and is especially associated with inadequate living conditions.

## INTRODUCTION

Trachoma is the leading cause of preventable infectious blindness in the world. It is endemic in some developing countries and belongs to the group of neglected tropical diseases, according to the World Health Organization^1^. It is an infectious disease caused by the bacterium *Chlamydia trachomatis* and its development is associated with poor socioeconomic and sanitation conditions^1^ ^-^ ^4^. 

Currently, it is estimated that 192 million people live in endemic areas and 1.6 million are affected by the disease, with 400,000 becoming irreversibly blind^5^. In the 1990s, under the leadership of the World Health Organization (WHO), the “Alliance for the Global Elimination of Trachoma by 2020” was established, to which Brazil is a signatory^4^ ^,^ ^5^. 

In Brazil, during the period known as the “economic miracle” (1950s to 1970s), there was a significant decrease in the detection of trachoma, suggesting that this disease was no longer a public health problem, which led to the misguided belief that the disease had been eradicated^6^ ^,^ ^7^. However, epidemiological research shows that trachoma persists in the country as a public health problem, and should be considered as a possible diagnosis when patients present with acute and/or chronic conjunctival inflammation^8^ ^-^ ^12^. 

In the last national survey carried out in 1,156 municipalities, in all regions of Brazil and involving 119,531 students, a prevalence of 5% of trachoma was observed. Some locations had a higher prevalence, such as 7.9% in Acre and 8.7% in Ceará. Considering the variables evaluated, significant differences were found between students of rural and urban origin and between children under five years old and older age groups. There was a higher prevalence among students from rural areas and lower age groups. There was no significant difference between men and women^9^.

A survey of students from the state of Roraima revealed an average prevalence of 4.5%, and some municipalities had a prevalence greater than 10%. The researchers conducted the study with 6,986 elementary and high school students, with an average age of 9.2 years. Among the variables studied, a significant difference was found between students from rural areas and aboriginal communities in relation to those from urban areas^10^. In a study carried out in the countryside region of São Paulo, there was greater involvement of students living in suburbs and intermediate regions of the city compared to those residing in the central region^11^.

The Jequitinhonha Valley, located in the Northeast region of Minas Gerais, is considered one of the poorest regions of Brazil, and there are no studies with a representative sample addressing the prevalence of trachoma in students in this region, where a considerable contingent of people live in rural areas. Therefore, this research aimed to determine the prevalence of trachoma and associated factors among elementary school students in the Jequitinhonha Valley, Minas Gerais.

## METHODS

This is a cross-sectional study, whose target population consisted of elementary school students. The study setting was the Jequitinhona Valley, Northeast of Minas Gerais. The region has 29 municipalities and is one of the poorest in Brazil^13^. 

For sample selection, we used the cluster sampling technique, where each of the elementary schools was considered as one cluster. School selection was done randomly by drawing lots. Lastly, a group of students was drawn, where all the students were eligible for the study. 

For the sample size calculation, a confidence level of 95%, an estimated prevalence of 6.0%, and a sampling error of 2.5% were considered. The value found was multiplied by a correction factor for the design effect of 1.5, considering cluster sampling. 

Prior to data collection, the selected schools were informed of the study and supported its implementation. The students and their legal guardians, after formal communication and agreeing to participate in the research, signed the terms of consent and answered the questionnaire containing information on socioeconomic aspects about the participants and housing conditions.

Data collection was conducted from January to December 2018 by one of the researchers, an ophthalmologist with experience in trachoma. 

Clinical examination was performed using a 2.5D magnifying glass and an eyelid eversion maneuver to identify the characteristic lesions. Cases with clinical signs were considered positive according to the WHO criteria, which suggests that diagnosis should be given when there are at least two of the following clinical signs:

- follicles in the upper tarsal conjunctiva
- limbal follicles, also called Herbert’s pits
- typical conjunctival scar, which may be vertical and/or horizontal
- upper limb *pannus*, which corresponds to the invasion of neo-formed blood vessels

All students identified as having a positive result underwent conjunctival scraping. The material collected was sent for laboratory analysis and then individuals were referred for treatment and free monitoring by one of the researchers, an ophthalmologist. In addition, subjects were also offered eye evaluation and treatment as needed. Direct immunofluorescence was the test used to confirm the presence of the bacteria in the community. This test, although not very sensitive, has high specificity for trachoma, in addition to practicality and low cost. The presence of five or more elementary bodies in the conjunctival scrape was considered a positive result.

All children and adolescents regularly enrolled in elementary school were included in the study. Children and adolescents who were not at school on the data collection day, and/or did not present the completed socioeconomic questionnaire, were excluded from the study. 

Data were processed with the support of the Statistical Package for the Social Sciences software, Version 22.0 (IBM Corp., Armonk, N.Y., USA). Descriptive analyses were initially performed and then bivariate analyses were conducted using the chi-square test between the characteristics of the study group and the presence of trachoma. Variables that were statistically associated with a level of ≥20% (p <0.20) were evaluated jointly by binary logistic regression.

All ethical aspects were considered and the procedures followed were in accordance with the ethical standards of the Ethics Committee of the Universidade Estadual de Montes Claros (UNIMONTES), and in accordance with the principles of Declaration of Helsinki, 1964. Participants and their families were assured of anonymity and confidentiality.

## RESULTS

A total of 478 children and adolescents, aged 7 to 16 years, were examined. Most of the students evaluated were female (60.5%) and the mean age was 11.5 (Standard Deviation [SD] 2.4) years. Regarding the type of school and location, public schools and schools in the central regions of urban areas predominated. Thirty cases clinically diagnosed as trachoma were identified, corresponding to a prevalence of 6.3%, all of which were classified as follicular inflammatory trachoma. The direct immunofluorescence laboratory test detected 36.6% positivity. Further details are described in Table 1. 

**TABLE 1:** Demographic and social characterization of students in the Jequitinhonha Valley region, Minas Gerais, Brazil, 2018.

Variable	(n)	(%)
Gender		
Male	189	39.5
Female	289	60.5
Age		
≤10 years	167	34.9
11 to 13 years	204	42.7
>13 years	107	22.2
Current school grade		
1^st^ to 5^th^ grade	193	40.4
6^th^ to 9^th^ grade	285	59.6
School type		
Municipal	179	37.4
State	299	62.6
School location		
Urban area (central region)	253	52.9
Urban area (suburban region)	93	19.5
Rural area	132	27.6
Number of people residing in the household		
≤4	159	33.3
5-7	278	58.2
>7	41	8.6
Is there a maid or cleaner in the household?		
Yes	39	8.2
No	439	91.8
Is the mother employed?		
Yes	224	48.5
No	240	44.2
No information	16	7.4
Is the father employed?		
Yes	273	65.6
No	96	23.5
No information	15	11.0
Presence of trachoma		
Yes	30	6.3
No	448	93.7

Table 2 presents the characterization of the students’ housing conditions. Almost all respondents lived in their own homes (83.3%) which were finished (86.8%). Approximately one-third of households were not provided with a sewage system (33.7%). Although almost all families had access to electricity, 20.5% did not have an electric shower. This information is important for assessing socioeconomic and hygiene conditions.

**TABLE 2:** Characterization of the residences of students from the Jequitinhonha Valley region, Minas Gerais, Brazil, 2018.

Variable	(n)	(%)
Property type		
Own property	398	83.3
Rented property	67	14.0
Living with relatives	13	2.7
Property Characteristics		
Finished house	415	86.8
Unfinished house*	63	13.2
Sewage system		
Yes	317	66.3
No	161	33.7
Bathroom inside the house		
Yes	468	97.9
No	10	2.1
Electric shower		
Yes	380	79.5
No	98	20.5
Water		
Yes	476	99.6
No	2	0.4
Electricity		
Yes	471	98.5
No	7	1.5
Roof		
Concrete slab	79	16.5
Ceramic tiles	388	81.2
Others	11	2.3
Flooring		
Earthen/Simple cement	107	22.4
Parquet/Hardwood	13	2.7
Ceramic/Slate/Porcelain	346	72.4
Others	12	2.5
Number of bedrooms		
≤2	140	29.3
>2	338	70.7

(*) No plastering, painting, flooring, and unfinished bathrooms.

Table 3 presents the results of bivariate analyses for the association between demographic and social characteristics and the presence of trachoma among the students. All covariates related at p <0.20 to the outcome were jointly evaluated by logistic regression. 

**TABLE 3:** Association between demographic and social variables and presence of trachoma in students living in the Jequitinhonha Valley, Minas Gerais, Brazil, 2018 (bivariate analysis).

Variables	Trachoma				p-value*
	Yes		No		
	(n)	(%)	(n)	(%)	
Gender					0.661
Male	13	43.3	176	39.3	
Female	17	56.7	272	60.7	
Age					0.558
≤10 years	9	30.0	158	35.3	
>10 years	21	70.0	290	64.7	
Current school grade					0.965
1^st^ to 5^th^ grade	12	40.0	181	40.4	
6^th^ to 9^th^ grade	18	60.0	267	59.6	
Number of people residing in the household					0.108
≤4	14	46.7	145	32.4	
>4	16	53.3	303	67.6	
Type of school					0.008
Municipal	18	60.0	161	35.9	
State	12	40.0	287	64.1	
School location					<0.001
Rural area	18	60.0	114	25.4	
Urban area	12	40.0	334	74.6	
Is there a maid or cleaner in the household? (Fisher’s exact test)					0.497**
No	29	96.7	410	91.5	
Yes	1	3.3	38	8.5	
Is the mother employed?					0.437
No	18	60.0	236	52.7	
Yes	12	40.0	212	47.3	
Is the father employed?					0.898
No	7	23.3	100	22.3	
Yes	23	76.7	348	77.7	

(*) Chi-square test; (**) Fisher’s exact test.

Table 4 presents bivariate analyses results. Similar to the analysis of demographic and social data, all covariates related at p <0.20 were jointly assessed by logistic regression. 

**TABLE 4:** Association between housing characteristics and presence of trachoma in students living in the Jequitinhonha Valley, Minas Gerais; 2018 (bivariate analysis).

Variables	Trachoma				p-value*
	Yes		No		
	(n)	(%)	(n)	(%)	
Type of property					0.528
Rented/Living with relatives	4	13.3	80	17.9	
Own property	26	86.7	368	82.1	
Property Characteristics					0.024
Finished house	8	26.7	55	12.3	
Unfinished house***	22	73.3	393	87.7	
Sewage system					0.001
No	25	83.3	169	37.7	
Yes	5	16.7	279	62.3	
Bathroom inside the house (Fisher)					0.125**
No	2	6.7	8	1.8	
Yes	28	93.3	440	98.2	
Number of bedrooms					0.117
≤2	5	16.7	135	30.1	
>2	25	83.3	313	70.7	
Flooring					0.053
Earthen/Simple Cement	11	36.7	96	21.4	
Others	19	63.3	352	78.6	
Roof					0.320
Ceramic tiles	27	90.0	372	83.0	
Concrete slab	3	10.0	76	17.0	
Electric shower					0.692
No	7	23.3	91	20.3	
Yes	23	76.7	357	79.7	

(*) Chi-square test; (**) Fisher’s exact test. (***) No plastering, painting, flooring, and unfinished bathrooms.

Table 5 presents the final model of logistic regression analysis. The variables that were associated with the presence of trachoma were: living in a residence with no finishing, living in a residence without sewage system coverage, and studying in a rural area.

**TABLE 5:** Factors associated with the presence of trachoma in students living in the Jequitinhonha Valley, Minas Gerais; 2018 (multiple analysis by logistic regression).

Variable	p-value	OR (95% CI)*
Property Characteristics	0.027	
Unfinished house**		2.27 (1.12-6.48)
Finished house		1.0
Sewerage system	<0.001	
No		9.49 (3.52-25.60)
Yes		1.0
School location	0.002	
Rural area		3.37 (1.53-7.35)
Urban area		1.0

(*) OR: Odds ratio; CI: Confidence interval. (**) No plastering, painting, flooring, and unfinished bathrooms.

## DISCUSSION

The prevalence of trachoma among students of the Jequitinhonha Valley was 6.3%, which is slightly above the value of 5% recommended by WHO as indicative that the disease under control^1^ ^-^ ^3^ ^,^ ^7^. The existence of trachoma in a population is an indicator of precarious living conditions, which also suggests the need for socioeconomic improvements^1^ ^-^ ^3^ ^,^ ^7^ Considering the vulnerable conditions of the evaluated population, this result should be taken as a warning sign since diagnosis may be neglected by health professionals, as there are few reports of the disease in the region.

In a survey conducted on 119,531 students and 1,156 municipalities in all regions of the country, an average trachoma prevalence of 5% was identified. In Minas Gerais, the study recorded an average prevalence of 4.8%, which is slightly below the results reported in the present study^9^. This can be explained by the size and socio-economic differences between the populations studied. Similar to the current study, a significant difference was found between urban and rural students, the latter being most affected, while no significant difference was detected between males and females. However, unlike the present study, a higher prevalence was found among children under five years old^9^. This difference can be explained by the size and differences in the age groups of the populations studied.

A study on elementary school students from the state of Roraima, revealed an average prevalence of 4.5%, a value below that found in this research. This can be explained by the similar socioeconomic characteristics among the populations studied. Among the studied variables, similar to this study, a significant difference between rural and urban area students was found and there was no significant difference between genders and between age groups^10^.

In a survey conducted on students from the city of Turmalina (in Minas Gerais), researchers found a trachoma prevalence of 4.7%, slightly below the value found in the current study. This can be explained by the fact that this city, although part of the Jequitinhonha Valley, has better socioeconomic indices than most cities in the region. However, there was a statistically significant difference between rural and urban area students and no difference between genders^12^, which corroborated our findings.

The variables that were associated with the presence of trachoma in students, after multiple analysis, indicate mostly social exclusion, which is characterized by poverty and difficulty in accessing goods and services. Unfinished housing conditions probably reflect the family's financial struggle and, consequently, a low social and economic level. 

The lack of adequate water waste treatment also denotes lack of access to social assets and poor housing conditions. In a systematic review study, Stocks et al.^14^ also evidenced the association between the presence of trachoma and poor sanitation. Regarding school location, a higher prevalence was identified in the rural area compared to the urban area, which is in accordance with the literature^9^ ^,^ ^10^ ^,^ ^12^. This finding might be linked to socioeconomic and sanitation conditions, which are usually worse in rural areas compared to urban regions.

Although age and gender were associated in other studies with trachoma, they were not statistically associated in the present study. Regarding age, previous studies verified a higher prevalence in younger children compared to older children^9^ ^,^ ^10^ ^,^ ^15^. This finding can be attributed to differences in sample size and the age group studied. Regarding gender, some studies showed a higher prevalence among women compared to men^15^ ^,^ ^16^ ^,^ ^17^. Some authors believe this may be due to females showing more affective behavior among themselves than males^16^ ^,^ ^18^.

The other variables studied, such as the presence of water, light, or showers, were not associated, which differed from other studies^15^ ^,^ ^17^ ^,^ ^19^. These variables are not likely to have great relevance in the spread of the disease in the studied population.

Herein, the WHO criteria were used in the clinical diagnosis, which considers the clinical state with well-established signs of the disease as a positive result. Therefore, this could be a limiting factor to this study, as the identification of early cases might have been hindered. Moreover, a higher prevalence would be expected in preschool children, who were not evaluated.

Based on this study, considering that the presence of trachoma can be seen as an indicator of a population’s socioeconomic conditions, the Jequitinhonha Valley needs government policies aimed at improving this situation, including basic sanitation and improvement in living conditions of the population, especially in rural areas.
